# Comparison of effectiveness and cost of patent ductus arteriosus device occlusion versus surgical ligation of patent ductus arteriosus

**DOI:** 10.12669/pjms.324.10048

**Published:** 2016

**Authors:** Arif Zulqarnain, Muhammad Younas, Tariq Waqar, Ahsan Beg, Touseef Asma, Mirza Ahmad Raza Baig

**Affiliations:** 1 Dr. Arif Zulqarnain, MBBS, FCPS Paediatrics. Fellow Paeds Cardiology, Ch. Pervaiz Elahi Institute of Cardiology Multan, Pakistan; 2 Dr. Muhammad Younas, FCPS Paediatrics, FCPS Paeds Cardiology. Assistant Professor and Head of Paeds Cardiology Department, Ch. Pervaiz Elahi Institute of Cardiology Multan, Pakistan; 3 Dr. Tariq Waqar, FCPS Surgery, FRCS. Associate Professor and Head of Paeds Cardiac Surgery Department, Ch. Pervaiz Elahi Institute of Cardiology Multan, Pakistan; 4 Dr. Ahsan Beg, MBBS, FCPS Paediatrics, FCPS Paeds Cardiology. Assistant Professor Paeds Cardiology, Ch. Pervaiz Elahi Institute of Cardiology Multan, Pakistan; 5 Dr. Touseef Asma, MBBS, FCPS Paediatrics, FCPS Paeds Cardiology. Senior Registrar Paeds Cardiology, Ch. Pervaiz Elahi Institute of Cardiology Multan, Pakistan; 6 Mr. Mirza Ahmad Raza Baig, B.Sc Hons Cardiac Perfusion Technology. Ch. Pervaiz Elahi Institute of Cardiology Multan, Pakistan

**Keywords:** Surgical ligation, Patent ductus arteriosus

## Abstract

**Objectives:**

Comparison of effectiveness and cost of transcatheter occlusion of patent ductus arteriosus (PDA) with surgical ligation of PDA.

**Methods:**

This retrospective comparative study was conducted in the pediatric cardiology department of Ch. Pervaiz Elahi Institute of Cardiology Multan, Pakistan. Data of 250 patients who underwent patent ductus arteriosus (PDA) closure either surgical or trans-catheter closure using SHSMA Occluder having weight >5 kg from April 2012 to October 2015 were included in this study. SPSS version 20 was used for data analysis. Quantitative variables were compared using independent sample t-test. Chi-square test and fishers exact was used for qualitative variables. P-value <0.05 was considered statistically significant.

**Results:**

There were one hundred and twenty (120) patients who underwent transcatheter occlusion of PDA using SHSMA occluder (PDA Device Group) and one hundred and thirty (130) patients who underwent surgical ligation of PDA (Surgical Group). Incidence of residual shunting was two (1.5%) in surgical group and 0 (0.0%) in PDA Device group for one month follow up period. There were 4 (3.1%) major complications in surgical group. The rate of blood transfusions were high in surgical group (p-value 0.04). Hospital stay time was significantly less in PDA Device group (P-value <0.001). Total procedural cost was 110695+1054 Pakistani rupees in PDA Device group and 92414+3512 in surgical group (p-value <0.001). The cost of PDA device closure was 16.52% higher than the surgical ligation of PDA. There was no operative mortality.

**Conclusion:**

The transcatheter closure of PDA is an effective and less invasive method as compared to the surgical ligation. There is a lower rate of complications and the cost is not much high as compared to surgical PDA ligation.

## INTRODUCTION

The ductus arteriosus is responsible for carrying 55-60% of total fetal output during intrauterine life and is usually closed within 2 to 3 weeks of age after birth.^1^ The persistence of blood flow through this channel after this age is a defect, which is responsible for 7-11% of all congenital cardiac defects.^2^,^3^ The incidence reaches to 80% in premature neonates having weight less than 1.2 kg^1^. Persistence of Patent Ductus Arteriosus (PDA) generates a picture of pulmonary over-circulation, which intern results in congestive Cardiac failure with low weight gain and repeated pulmonary infections and in the long run pulmonary arterial hypertension.^1^ Early PDA closure prevents the development of complications e.g. cardiac failure, pulmonary arterial hypertension and infections.^1^-^4^

Surgical ligation of PDA was 1^st^ described in 1930s through lateral thoracotomy with good results.^5^ From past two decades, closure of PDA with latest generation devices has been performed with very good results in various age groups.^6^ PDA closure with Amplatzer duct occluder (ADO) has proved an excellent therapeutic option.^7^-^10^ The high cost of this device and other devices as compared to surgical ligation of PDA has limited their widespread use. Surgical ligation of PDA is a widely accepted technique and is still being used in many centers in Pakistan. Sun WF et al., have shown excellent outcomes of PDA closure using the SHSMA occlude.^11^ This occluder is very cheap in price as compared to the ADO and other devices. But we do not find any trial comparing the cost and effectiveness of SHSMA occluder versus surgical ligation. So we planned this study to compare the cost difference and effectiveness of Transcatheter PDA closure using SHSMA occluder versus surgical ligation of PDA in a tertiary care center.

## METHODS

This retrospective comparative study was conducted in the pediatric cardiology department of Ch. Pervaiz Elahi Institute of Cardiology Multan, Pakistan. Data of 250 patients who underwent patent ductus arteriosus (PDA) closure either surgical or transcutaneous closure from April 2012 to October 2015 with a follow up period of one month was retrieved from the data base system of cardiac surgery and pediatric cardiology department of the hospital. Regarding inclusion criteria following patients were included in this study; patients having weight more than 5 kg (no upper limit was set), age ≥1 years to 60 years and diagnosed of having moderate to large sized PDA (having minimum duct diameter of 4-16 mm pre-procedurally). While patients with incomplete records, having window like PDA, or any other cardiac or non-cardiac morbidity that could affect the procedural outcome and patients with congestive heart failure at the time of procedure were excluded from this study. The choice of surgical or transcatheter closure was mostly based on the patients or the family preferences. No informed consent was taken because of the retrospective nature of the study. Ethical approval was taken from the department of academic affairs of the hospital before starting the research work.

### Technique of PDA Closure

The SHSMA occluder device was made off Shanghai Shape Memory Alloy Company. The transcatheter occlusion was performed in cardiac catheterization laboratory of the hospital under local anesthesia. The occluder selected diameter was about 2-4 mm larger than the minimum duct size. Antibiotic prophylaxis was given in every patient.

Surgical ligation was performed under general anesthesia in the operating room through lateral thoracotomy. Anesthesia was induced using intravenous administration of midazolam, Fentanyl or morphine and atracurium bromide. The doses of these drugs were adjusted according to the patient’s response. Ligation was done by triple ligation of the patent ductus arteriosus. Tube thoracostomy was done in every patient. Antibiotic therapy was given until removal of tube. Follow up data regarding echocardiography and physical examination was taken for a period of one month after surgery. Complications related with either procedure were recorded for each procedure. Post-procedural echocardiography was performed next day after the procedure and after one month of procedure using Vivid-7 system (GE Medical System, Milwaukee, WI, USA).

Total hospital charges were calculated for every patient for both groups. Routine follow-up cost was not added in this cost. Only the cost related to the procedure was included in this study. The total cost was calculated by adding hospital charges, consultant charges, private room charges, device cost and other equipment used during insertion of device, laboratory investigation charges, blood transfusion charges and post-op echocardiographic charges.

SPSS version 20 was used for data analysis. Quantitative variables were compared using independent sample t-test. Chi-square test and fishers exact was used for qualitative variables. P-value <0.05 was considered statistically significant.

## RESULTS

There were one hundred and twenty (120) patients who underwent transcatheter occlusion of PDA using SHSMA occluder (PDA Device Group) and one hundred and thirty (130) patients who underwent surgical ligation of PDA (Surgical Group). Surgical group patients were little older as compared to PDA Device group. There were more female patients in this study. There were 61.7% female patients in PDA Device group and 54.6% in surgical group. Body weight, pulmonary hypertension and minimum duct size all were almost same between the two groups. Incidence of residual shunting was 2 (1.5%) in surgical group and 0 (0.0%) in PDA Device group at a follow up period of one month.

**Table-I:** Comparison of Pre-operative Variables.

	PDA Device Group	Surgical Group	P-value
Number of Patients	120	130	
Age (year)	8.15+7.8	9.79+7.5	0.09
Gender Female (%)	74 (61.7)	71 (54.6)	0.26
Body Weight (kg)	22.86+16.73	20.16+20.13	0.25
Pulmonary Hypertension (%)	17 (14.2)	17 (13.1)	0.80
Minimum Duct Size (mm)	4.28+1.68	4.20+1.56	0.69
LV Ejection Fraction (%)	64.97+6.02	63.41+6.19	0.08

Continuous variables were presented as mean+SD. LV= Left Ventricle.

One patient in PDA Device group underwent surgical PDA closure due to device slip and removal of the device was done in the operating room. There were 4 (3.1%) major complications in surgical group two of which were PDA tear during the procedure, which significantly lengthen the procedure. All patients in surgical group required general anesthesia while no patient in PDA Device group underwent general anesthesia. There were more patients in surgical group who required blood transfusion during the surgical procedure (p-value 0.04). Hospital stay time was significantly less in PDA Device group, it was only 1.58+0.69 days in PDA Device group versus 4.36+1.17 days in surgical group with p-value of <0.001. Total procedural cost was one lac ten thousand Pakistani rupees (exact) in PDA Device group and about ninety three thousand rupees (exact) in surgical group (p-value <0.001). There was no mortality within the period of one month in each group.

## DISCUSSION

The timely closure of PDA leads to normal life expectancy of patient equal to normal population. Gross and Hubbard 1^st^ reported surgical ligation of PDA in 1938 after that surgical treatment of PDA remained the gold standard treatment for many decades.^12^ Since the 1^st^ report of PDA closure by trans-catheter method by Porstmann et al., many devices such as Siderisbutton device, Rashkind double umbrella, coil closure, Amplatzer duct occluder and SHSMA occluder have been used to occlude PDA. These devices are used to occlude small to large sized PDAs.^13^,^14^ This study was conducted to compare the clinical outcomes of patients who underwent PDA closure either surgical or with SHSMA occluder device and to see the effectiveness and cost effectiveness of both procedures.

**Table-II:** Comparison of Operative Outcomes.

	PDA Device Group	Surgical Group	P-value
Residual Shunting (%)	00 (0.0)	02 (1.5)	0.17
Repeat of Procedure (%)	01 (0.8)	00 (0.0)	0.33
Major Complication (%)	01 (0.8)	04 (3.1)	0.20
General Anesthesia (%)	00 (0.0)	130 (10)	<0.001
Requirement of Blood (%)	01 (0.8)	07 (5.4)	0.04
Hospital Stay (days)	1.58+0.690	4.36+1.17	<0.001
Total Cost (Rs.*)	110695+1054	92414+3512	<0.001

* Rs. = Pakistani rupees, Cost of SHSMA occluder is 72000 Pakistani Rupees.

The SHSMA occluder device closure was associated with a least number of complications according to the results of this study. There was only one incidence that was device slip in one patient that required surgical intervention. The incidence of residual shunting was nil in the follow up period of one month. The incidence of residual shunting in surgical group was 1.5% in this study. Many other studies have reported that the incidence of residual shunting in surgical closure of PDA varies from 1.5 to 23%.^8^,^14^,^15^

Hospital stay time in our study was significantly less in PDA Device group. Many other studies have reported a shorter hospital stay time in patients who underwent transcatheter closure. The shorter hospital stay in PDA Device group was due to less invasive nature of the procedure. Chen et al. and Jeong el al., reported a longer hospital stay time in low income countries in surgical PDA device closure group,^14^,^16^ but in our study the hospital stay time was shorter and was comparable with that of modern countries.^17^,^18^

The cost of PDA device closure in this study was 16.52% higher than the surgical ligation of PDA. But device closure was associated with least number of complications and less hospital stay. It also bypasses the requirement of general anesthesia and blood transfusions, and saves the patients from their deleterious effects. And especially there was no mark on the skin as in case of surgical closure. And the cost was not much high as compared to surgical group, there was a difference of only Rs. 17,000 to 18,000 (16.52%) per case which can be compensated by the patient easily because longer hospital stay is associated with many other expenses of food and other things. So we concluded that PDA closure using SHSMA occluder device was not much expensive as compared to the surgical PDA closure.

## CONCLUSION

The transcatheter closure of PDA is an effective and less invasive method as compared to the surgical ligation. There is a lower rate of complications and the cost is not much high as compared to surgical PDA ligation.
